# Effects of state anxiety on gait: a 7.5% carbon dioxide challenge study

**DOI:** 10.1007/s00426-020-01393-2

**Published:** 2020-07-31

**Authors:** Angela S. Attwood, Casimir J. H. Ludwig, Ian S. Penton-Voak, Jade Poh, Alex S. F. Kwong, Marcus R. Munafò

**Affiliations:** 1 MRC Integrative Epidemiology Unit, University of Bristol, Bristol, UK; 2 UK Centre for Tobacco and Alcohol Studies, School of Experimental Psychology, University of Bristol, Bristol, UK; 3 School of Experimental Psychology, University of Bristol, 12a Priory Road, Bristol, BS8 1TU UK; 4 School of Geographical Sciences, University of Bristol, Bristol, UK; 5 Bristol Vision Institute, University of Bristol, Bristol, UK

## Abstract

We used the 7.5% carbon dioxide (CO_2_) model of anxiety induction to investigate the effects of state anxiety on normal gait and gait when navigating an obstacle. Healthy volunteers (*n* = 22) completed a walking task during inhalations of 7.5% CO_2_ and medical air (placebo) in a within-subjects design. The order of inhalation was counterbalanced across participants and the gas was administered double-blind. Over a series of trials, participants walked the length of the laboratory, with each trial requiring participants to navigate through an aperture (width adjusted to participant size), with gait parameters measured via a motion capture system. The main findings were that walking speed was slower, but the adjustment in body orientation was greater, during 7.5% CO_2_ inhalation compared to air. These findings indicate changes in locomotor behaviour during heightened state anxiety that may reflect greater caution when moving in an agitated state. Advances in sensing technology offer the opportunity to monitor locomotor behaviour, and these findings suggest that in doing so, we may be able to infer emotional states from movement in naturalistic settings.

**Electronic supplementary material:**

The online version of this article (10.1007/s00426-020-01393-2) contains supplementary material, which is available to authorized users.

## Introduction

Abnormal gait is common in a number of psychiatric disorders comprising motor deficits, such as Parkinson’s disease. However, changes in gait are also observed in other mental health conditions, including schizophrenia and depression (Sanders & Gillig, 2010). In fact, gait differences have been reported between normally functioning adults with high and low mood, with low mood individuals using reduced push-off force when walking (Sloman, Pierrynowski, Berridge, Tupling, & Flowers, 1987). The association between gait and mental health is unsurprising given that cognitive systems have been shown to play a role in locomotion and balance (Al-Yahya et al., 2011). Furthermore, Sanders and Gillig (2010) identify that as gait requires higher level brain systems, analysis of it can improve the understanding of psychiatric disorder. Taken together, these findings indicate that gait may be altered by psychological states, although the nature of these changes in response to different psychological states is unknown and warrants further investigation. In this study, we use a laboratory model of anxiety induction to investigate how gait may be altered in heightened anxious states.

Recent advances in ubiquitous sensing technology offer new opportunities to infer emotional and cognitive states from movement in naturalistic settings. To date, a few studies have examined the effects of acute anxiety on gait, despite anxiety being associated with a number of motor-related symptoms including increased muscle tension (Pluess, Conrad, & Wilhelm, 2009). It has been suggested that anxiety may contribute to freezing of gait in Parkinson’s disease (Martens et al., 2016). In healthy volunteers, one study (conference proceeding) has reported changes in walking velocity and stride length following anxiety induction using a psychological stressor (Long, Kovacs, & Acevedo, 2004). More recently, Johnson et al. (2019) examined the effect of threat on postural control, and found increases in the frequency and amplitude of postural sway under increased threat. To exploit emerging technologies to develop monitoring systems for anxiety-related conditions, further research is needed to identify the profile of movement associated with anxious states.

This study addresses this gap in the literature. Using the 7.5% carbon dioxide (CO_2_) inhalation model of anxiety induction, we compared gait parameters of individuals during anxious and non-anxious states in a within-subjects design. The CO_2_ procedure has substantial benefits over other anxiogenic challenges commonly used in experimental studies. Unlike many social stressors, which induce anxiety through anticipation of public speaking or tests, the inhalation of gas occurs during the task of interest, meaning that data are collected during peak anxiety. We have previously used this model to investigate the effects of state anxiety on a number of subjective and cognitive outcomes including emotional face processing (Attwood et al., 2017), face memory (Attwood, Catling, Kwong, & Munafo, 2015), language processing (Mattys, Seymour, Attwood, & Munafo, 2013), and threat perception (Garner, Attwood, Baldwin, James, & Munafo, 2011).

In this study, we used motion capture to measure gait variables (e.g., walking speed, body rotation when moving through an aperture), while participants inhaled 7.5% CO_2_ (anxiety condition) and medical air (control condition). We hypothesised that there would be differences on all measures between gas and air conditions. However, due to the paucity of research in this area, this research was exploratory and our hypotheses non-directional.

## Methods

The protocol for this study was pre-registered on the Open Science Framework (DOI 10.17605/osf.io/m4etx).

### Participants

Twenty-four healthy volunteers were recruited from staff and students of the University of Bristol and from the local area via participant email lists, posters, and word of mouth. Data from two participants were compromised due to incorrect positioning of poles. Therefore, data from 22 participants were used in all analyses. Participants were required to be in good physical and psychiatric health, and females were excluded if they were pregnant or breastfeeding. For a full list of exclusion criteria and screening procedures, see pre-registered protocol and Supplementary Information (Sect. 1.1). Participants were reimbursed £20 for taking part in the study. Informed consent was obtained from all individual participants included in the study.

### Study design

The study used a within-subjects design with a primary factor of gas (air, 7.5% CO_2_). To assess the subjective and physiological effects, self-report ratings of anxiety and mood, and physiological measurements of heart rate and blood pressure were taken after each inhalation.

### Measures and materials

**Fig. 1:**
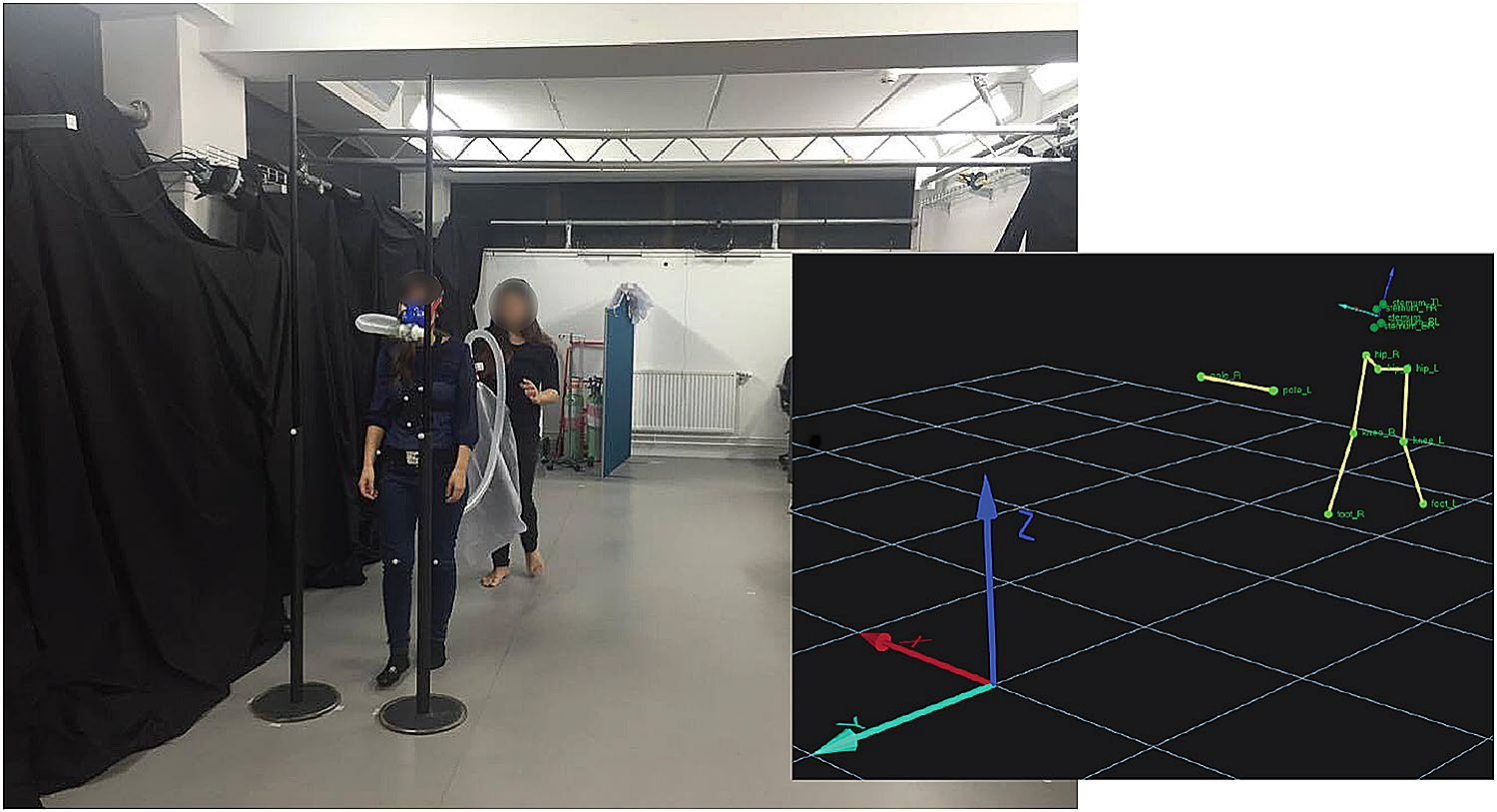
Illustration of the laboratory setup, locomotion task, the coordinate system, and marker set. The participant (one of the experimenters, for illustration purposes; actual participants wore shorts and/or tight-fitting clothing) walked through a right aperture. An experimenter, blind to the gas condition, walked behind the participant with a bag filled with 7.5% CO_2_ or medical air (placebo). Bags were filled by a second experimenter behind a partition (visible behind the experimenter on the left). The inset shows the global coordinate system and the marker set. We extracted body position and orientation from a set of four markers fixed to a plate that was worn around the chest (‘sternum plate’). This set of markers was defined as a rigid body (they never move relative to each other). Its local coordinate system is aligned with that of the global coordinate system (i.e., *x* pointing right, *y* pointing forward, *z* pointing up, shown in the bottom left of the inset). The horizontal yellow line connects two markers that were fixed to the poles, as shown in the image on the left

Gait measurements were collected using a 12-camera Qualisys optical motion capture system. Infrared reflective markers were fixed to the sternum, waist, knees, and feet of participants. The system samples x, y, and z position of each marker at a rate of 120 Hz (see Fig. 1). Over a total of 40 trials (20 per inhalation), participants were instructed to walk the length of the laboratory (a ~ 12 m central section of the room), navigating through an aperture and aiming for a traffic cone near the end of the room. The width of the aperture was tailored to each participant, using an aperture to shoulder ratio of 1.2. For more information on the size and positioning of apertures, see Supplementary Information (Sect. 1.2).

**Fig. 2:**
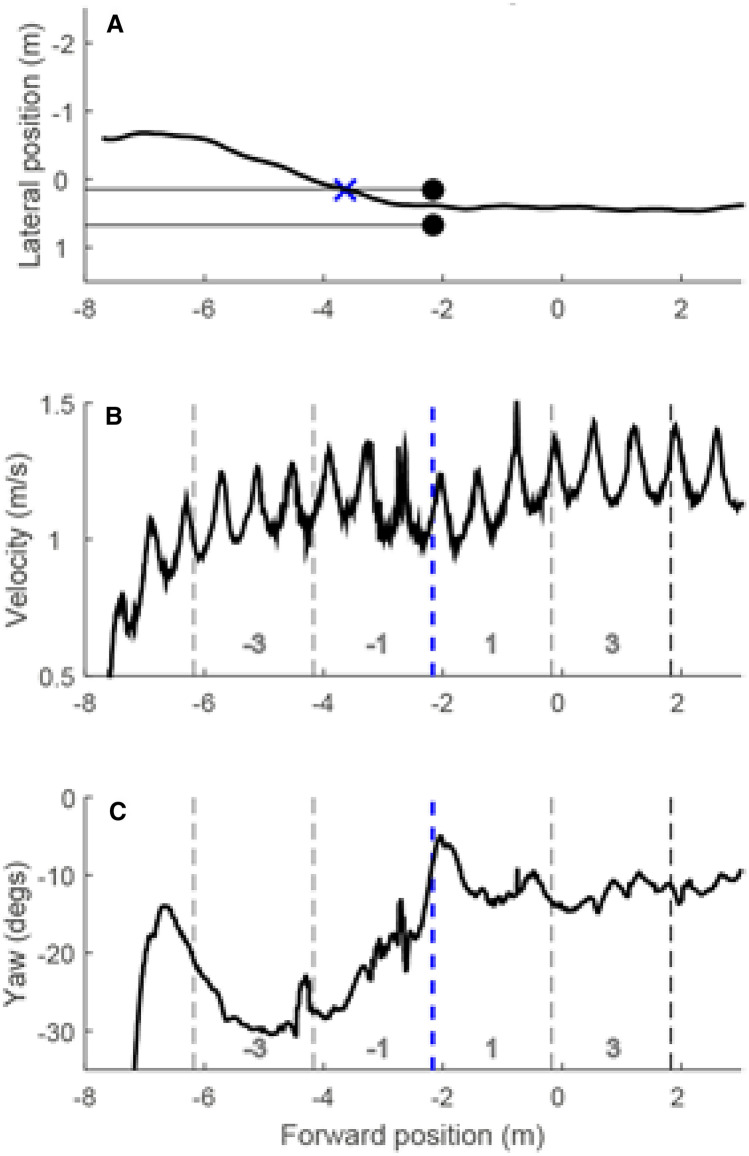
Raw data from a single trial. **a** Lateral position as a function of forward position; i.e., the walking trajectory viewed from a bird’s eye perspective. **b** Instantaneous walking speed as a function of forward position on the walkway. The velocity is combined for lateral and forward directions. **c** Body rotation around the vertical axis. The large change seen at the beginning of the trial can be attributed to the participant turning around to the walking direction. The consistently negative values indicated that the set of sternum markers from which we extracted body orientation was angled towards the right. Note that this orientation is a combination of the body orientation of the participant and the angle at which the plate with the sternum markers was placed on the chest

The gait-related dependent variables are described below. Figure 2 shows how each measure was operationalised using raw data from one participant during a single trial.

1. Entry point (Fig. 2a): as noted in the protocol, we intended to measure the distance from the aperture at which the participant deviated from straight ahead to heading towards the aperture (lateral deviation). However, in many cases, participants veered towards the aperture immediately. To accommodate this behaviour, a “corridor” was defined that was aligned to the lateral position of the poles. We measured the point at which the participant entered the corridor (i.e., crossed the lateral position of the inner pole). This point is marked by a cross in Fig. 2, panel a. We refer to this as “entry point” in this paper.
2. Walking speed (Fig. 2b) was analysed in four 2 m sections, with two sections before the aperture and two sections after. As noted in the protocol, we were interested in: (i) the aperture approach speed, measured in the 2 m section immediately before the aperture; (ii) baseline walking speed, measured in the final 2 m section after the aperture; (iii) the moment at which participants start to slow down before the aperture. A comprehensive analysis of walking speed across these four sections (with ‘section’ as a factor) allowed us to address all three measures in one analysis. Velocities were computed separately for *x* and *y* using three-point numerical differentiation, as implemented by the Matlab ‘gradient’ function. The *x* and *y* velocities were combined into a vectorial quantity, only the magnitude of which was kept. The combined velocity was then averaged over all the samples in each section.
3. Angular rotation (Fig. 2c) as the participant navigates through the aperture was also measured. As the aperture width was greater than the shoulder width, it was not necessary to pass through the aperture “sideways”. Nevertheless, we would expect participants to make adjustments to their body orientation in the approach to the aperture. To capture this critical “turn”, we computed the overall magnitude of turning behaviour in the same four walkway sections as used for our analysis of walking speed. We differentiated the yaw angle of the rigid body fixed to the sternum, with respect to forward position on the walkway. The resulting measure tells us how much the body orientation changed in a small spatial region. We ignored the sign of this measure and averaged the absolute change in turning angle within each walkway section as an overall index of the amount of body turning as a function of walkway position.

For further information on how gait variables were defined and measured, see Supplementary Information (Sect. 1.3).

Questionnaire measures included the Spielberger State-Trait Anxiety Inventory (STAI State, STAI Trait) (Spielberger, Gorsuch, Lushene, Vagg, & Jacobs, 1983), the Positive and Negative Affect Schedule (PANAS) (Watson, Clark, & Tellegen, 1988), and the Eysenck Personality Questionnaire Revised (EPQ-R) (Eysenck & Eysenck, 1991).

Physiological measures of heart rate (HR), systolic blood pressure (SBP), and diastolic blood pressure (DBP) were taken at baseline and after each inhalation (Omron M6 BP monitor, Omron Healthcare B.V., UK).

The gas mixtures were 7.5% CO_2_, 21% O_2_, N balance and medical air, which has same basic constituents as natural atmosphere (21% O_2_, N balance). The gases were administered through an oro-nasal face mask (Hans Rudolph Inc., USA), attached to a Douglas bag. Order of gas inhalation was counter balanced across participants. Gas was administered double-blind. One researcher, who had no direct interaction with the participant, filled bags of relevant gas, and passed to a second experimenter who ran the study, and was unaware of gas condition.

### Procedure

Participants completed a telephone screening prior to the study session to assess basic eligibility. On the day of testing, written informed consent was taken and further screening procedures were conducted. Baseline questionnaire measures were also completed (STAI-state, STAI-trait, PANAS, and ASI). Participants were then fitted with the face mask, which was connected to either the 7.5% CO_2_ gas or medical air as per the gas order counterbalancing.

Participants were asked to wear sport shorts or leggings to minimise extraneous motion of the markers. During each inhalation, participants completed 20 trials (i.e., walking the length of the laboratory navigating through the apertures). At the start of each trial, participants stood with their back to the walkway, while one of the researchers placed the poles at designated locations (per pre-determined aperture positioning sequence). The aperture positions were marked on the floor with tape to ensure that apertures could be moved quickly between trials. A researcher, who was blind to the gas condition, walked behind the participant carrying the Douglas bag. Subjective (STAI-state, PANAS) and physiological (SBP, DBP, HR) measures were taken after each inhalation. There was a wash-out period of 30 min between inhalations and there was a recovery period of 20 min after the second inhalation, during which participants completed the EPQ-R. After this, blood pressure (SBP, DBP) and heart rate were measured again to check that these had returned to baseline levels. The study session lasted approximately 2.5 h. At the end of the session, participants were reimbursed and debriefed.

### Statistical analysis

We calculated that a sample size of 24 participants, in a within-subjects design, would give us 80% power at an alpha level of 5% to detect an effect size of *dz* = 0.6 on walking speed between the gas and air conditions. Such an effect size is compatible with the effect of a relatively subtle visual manipulation on walking speed, reported by Ludwig et al. (2018).

As per the protocol, we conducted mixed-design ANOVAs with gas condition (7.5% CO_2_, air) as a within-subject factor and gas order (CO_2_/air, air/CO_2_) as a between-subjects factor. However, as noted in the Materials section, we created four spatial bins, which enabled us to integrate analysis of speed variables into a single analysis rather than analysing baseline (2–4 m. after the aperture), approach speed (-2-0 m. before the aperture) and the moment of speed adaptation, separately. As per the pre-registered protocol, we also computed Bayes factors as a measure of evidence strength. For brevity, only primary outcomes are reported here. Gas order effects and the results from the Bayesian analyses are given in Supplementary Information (Sect. 2).

Subjective measures of anxiety and mood (STAI-state, PANAS-positive, and PANAS-negative) and physiological measures (systolic blood pressure, diastolic blood pressure, and heart rate) after 7.5% CO_2_ and air were compared using t tests. Separate analyses to investigate order effects are presented in Supplementary Information (Sect. 2.1).

## Results

The data that form the basis of the results presented here are available from the University of Bristol Research Data Repository (http://data.bris.ac.uk/data/, 10.5523/bris.28qz26mc70f5n2vea0g9h0jwzn). Restrictions apply in accordance with participant consent.

### Participant characteristics

Participants (*n* = 22; 41% male) were aged between 19 and 33 years (*M* = 23, SD 3). Body mass index ranged between 18 and 28 (*M *= 23, SD 3). STAI Trait scores ranged between 25 and 42 (*M *= 33, SD 6). EPQ-R scores ranged between 2 and 16 (*M* = 8, SD 4) for psychoticism, 5 and 18 (*M* = 10, SD 5) for neuroticism, and 5 and 23 (*M* = 16, SD 5) for extraversion.

### Entry point

There was no evidence of a main effect of gas [*F*(1,20) = 0.003, *p *= 0.96, *η*_g_ = 0.00] or order [*F*(1,20) = 0.09, *p *= 0.76, *η*_g_ = 0.00]. There was weak evidence of a gas-by-order interaction [*F*(1,20) = 3.36, *p *= 0.08, *η*_g_ = 0.01]. As order effects were not of primary interest, more detailed information is provided on all order effects in Supplementary Information (Sect. 2.1).

### Walking speed

**Fig. 3:**
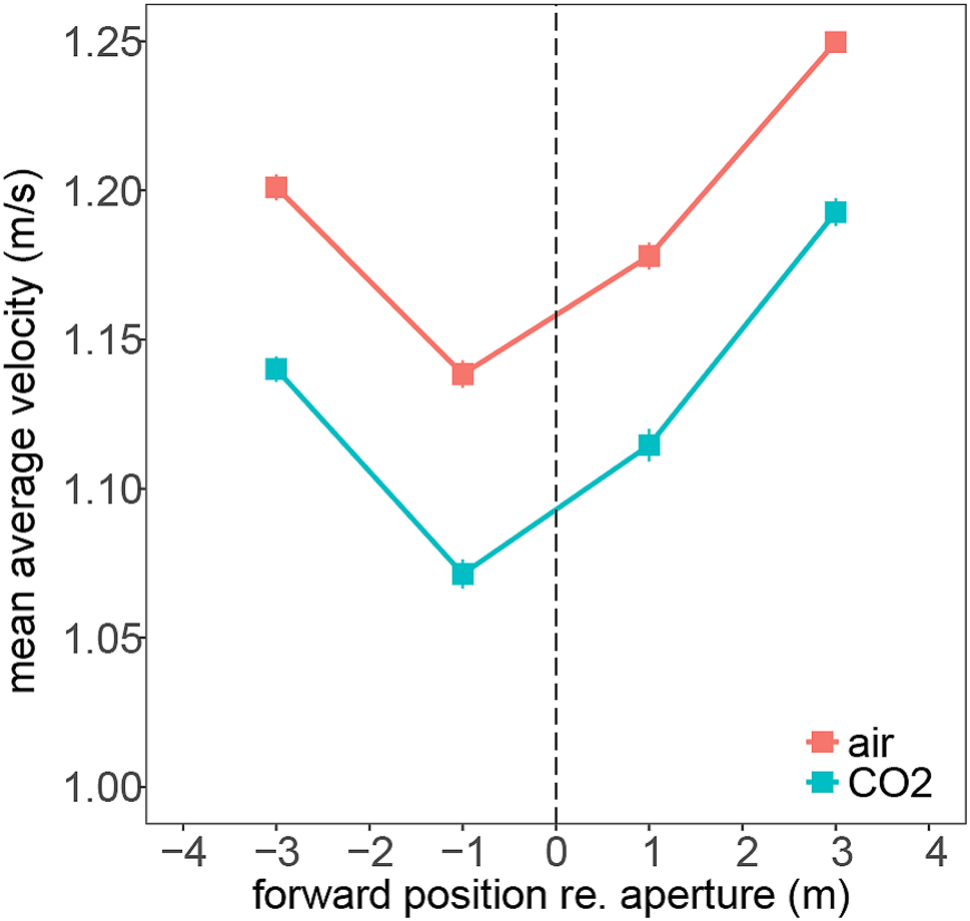
Walking speed. The aperture position is marked with the vertical dashed line. Error bars are within-subject standard errors of the mean and, in some conditions, smaller than the plotted symbols

Figure 3 shows walking speed in the four sections of the walkway. Overall, participants slowed down before the aperture and speed up afterwards. There was evidence of main effects of gas [*F*(1,20) = 20.29, *p *< 0.001, *η*_g_ = 0.05] and bin [*F*(3,60) = 36.73, *p *< 0.01, *η*_g_ = 0.09]. Adopting interpretation conventions, effect sizes for both factors were moderate (Ferguson, 2009). Bayes analysis provided further evidence against the null with a model that contained both gas and bin as predictors being “more likely” than the null model (a full model with all predictors and interactions had the highest Bayes Factor relative to the null model, from a total set of 18 possible models; see Supplementary Information, Sect. 2.2). As shown in Fig. 3, participants slowed down in the section immediately before the aperture (i.e., reduced approach speed), and were slower overall when inhaling CO_2_ compared to air.

There was also weak evidence of a main effect of order [*F*(1,20) = 4.14, *p *= 0.06, *η*_g_ = 0.15] and an order-by-condition interaction [*F*(1,20) = 5.81, *p *= 0.03, *η*_g_ = 0.02]. These are reported in more detail in Supplementary Information (Sect. 2.1).

There was no clear evidence of any other interactions: bin-by-order [*F*(3,60) = 1.70, *p *= 0.18, *η*_g_ = 0.00], condition-by-bin [*F*(3,60) = 0.80, *p *= 0.50, *η*_g_ = 0.00), and condition-by-bin-by-order [*F*(3,60) = 1.16, *p *= 0.33, *η*_g_ = 0.00].

### Body rotation (through an aperture)

**Fig. 4:**
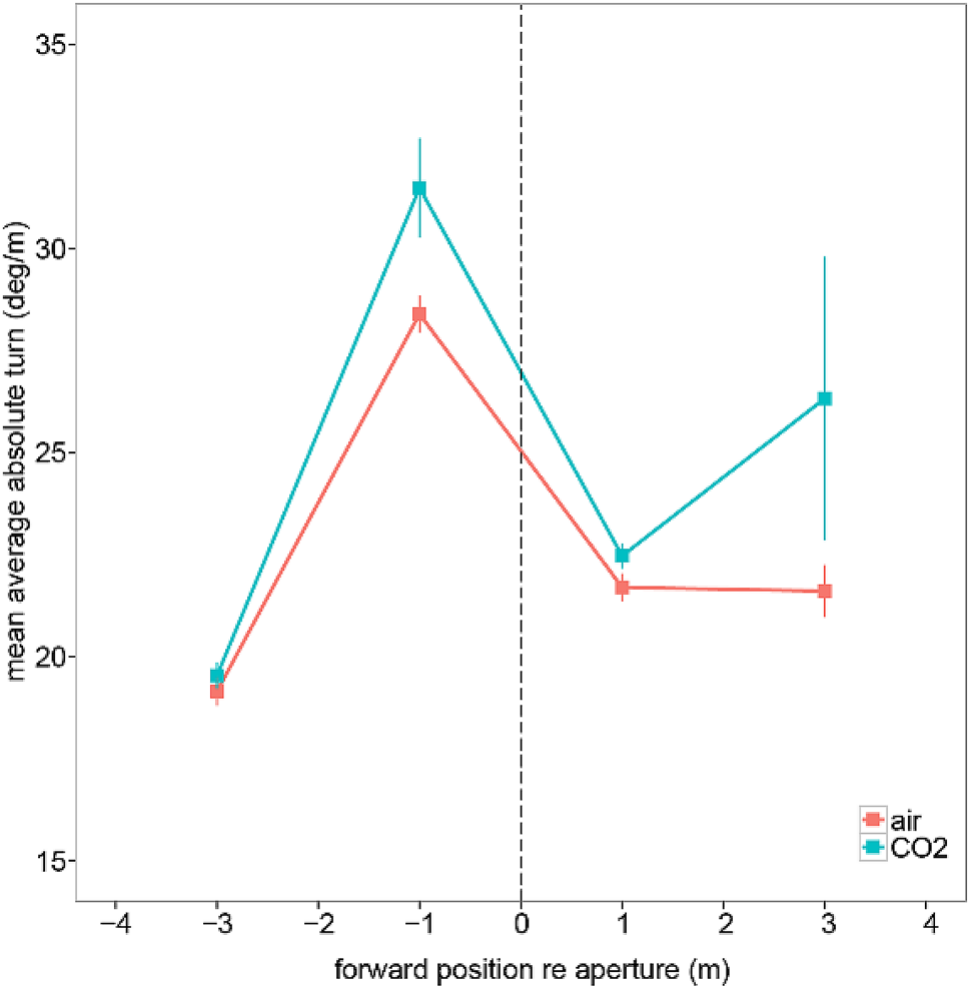
Turning behaviour. The aperture position is marked with the vertical dashed line. Error bars are within-subject standard errors of the mean

Figure 4 shows the overall magnitude of turning behaviour in each of the four positional bins. Substantial adjustment in body orientation occurred in the approach to the aperture. Note that this is a measure of overall change in body orientation and does not allow us to infer whether participants moved sideways or, indeed, “straightened up”. There was weak evidence of a main effect of gas condition [*F*(1,20) = 5.67, *p *= 0.03, *η*_g_ = 0.03], with greater body rotation in the CO_2_ condition, although the effect size is relatively small. There was also strong evidence of a main effect of bin [*F*(3,60) = 12.58, *p *< 0.001, *η*_g_ = 0.22], with a large effect of body rotation in the second (approach) bin. There was also evidence of a main effect of order [*F*(1,20) = 11.03, *p *= 0.003, *η*_g_ = 0.11] (see Supplementary Information for more details, Sect. 2.1). The Bayes factors were largely consistent with these inferences: the best model included gas and bin as predictor models (but not their interaction). However, in line with the small effect size reported above for the main effect of gas, the Bayes factor relative to the model without gas was only 1.61 (see Supplementary Information, Sect. 2.2).

There was no clear evidence of any interactions: bin-by-order [*F*(3,60) = 0.81, *p *= 0.49, *η*_g_ = 0.02], condition-by-bin [*F*(3,60) = 1.42, *p *= 0.25, *η*_g_ = 0.02], condition-by-order [*F*(1,20) = 1.22, *p *= 0.28, *η*_g_ = 0.01], and condition-by-bin-by-order [*F*(3,60) = 0.65, *p *= 0.59, *η*_g_ = 0.01].

### Manipulation check

State anxiety (STAI), negative affect (PANAS-negative), SBP, DBP, and HR were higher, and positive affect (PANAS-positive) was lower, after CO_2_ inhalation compared to air (see Table 1), confirming the validity of the anxiety manipulation.

**Table 1:** State anxiety, affect, and cardiovascular *t* test comparison data (*n* = 22)

	Mean difference (SD): CO_2_ vs air	95% CI	*P* value
STAI state	12.7 (7.5)	9.4 to 16.0	< 0.001
PANAS positive	− 3.9 (5.5)	− 6.4 to − 1.5	0.003
PANAS negative	4.9 (5.0)	2.7 to 7.1	< 0.001
SBP	11.4 (8.1)	7.8 to 15.0	< 0.001
DBP	2.6 (5.8)	0.6 to 5.2	0.045
HR	16.9 (11.6)	11.8 to 22.1	< 0.001

## Discussion

We found evidence that overall walking speed decreases during 7.5% CO_2_ inhalation compared to air. In addition, inhalation of 7.5% CO_2_ increased the degree of body rotation in the lead-up to the aperture. However, there was no evidence of an effect of gas on entry point and the start of speed adaption (i.e., in preparation for passing through the aperture). With regards to body rotation, the strongest evidence of a gas effect was in the second bin, in the approach to the aperture. The statistical evidence for a gas condition difference remained in the third bin, although was statistically weaker. We cannot identify the actual body rotation (in the world) from the measure used, because we do not have a reliable measure of the offset between the sternum plate and the global coordinate system of the room. The continued greater degree of rotation in the CO_2_ condition after the aperture may reflect compensatory adjustments due to greater (unwarranted) rotation before the aperture. On a related note, it could be that participants in the CO_2_ condition rotated their body more before the aperture, and in such a way that the body orientation was misaligned with the overall required trajectory. Again, in this case, the greater degree of turning after the aperture may reflect a compensatory response.

It is noteworthy that there was no evidence of an interaction between bin and condition for walking speed. This indicates that the effect of CO_2_ on walking speed when approaching the aperture (bin 2) was statistically equivalent to the effect on the overall, baseline speed (bin 4). Moreover, there was no evidence that the speed dropped at a different distance from the aperture in the CO_2_ condition, although our ability to identify such an effect is limited by the small number of spatial bins considered.

The decreases in walking speed during 7.5% CO_2_ inhalation may be due to participants reflecting more on their movement and exerting greater caution, which aligns with our other main finding of greater body rotation through the aperture. While there is little research on walking behaviour during anxious states, a review of the effects of state anxiety on perceptual–motor tasks identifies that anxiety may make individuals less accurate in their movements or require more time to execute them accurately (Nieuwenhuys & Oudejans, 2012). Using a different threat paradigm, Johnson et al. (2019) reported that threat was associated with changes in postural sway but also greater self-reported attention to movement and engagement with self-regulatory strategies. This aligns with our speculative explanation of the rotation effects being driven by increased caution in movement. Explicit monitoring theories that explain pressure-related performance failures argue that anxiety creates an attentional shift to internal factors, whereby there is increased conscious focus on the skill or behaviour that has otherwise become automated (Beilock & Carr, 2001; Pijpers, Oudejans, & Bakker, 2005). In the case of these data, this would suggest more conscious processing of gait behaviour during 7.5% CO_2_ inhalation, which in turn leads to a greater degree of caution. Here caution is expressed as lower walking speed and excessive postural adjustments when moving through an aperture.

There was evidence of order effects and interactions for several outcomes (see Supplementary Information, Sect. 2, for more detail). Generally, order effects are common with within-subject designs (gas condition), where we often observe stronger gas effects in the CO_2_/air order group. We believe that this can be attributed to some degree of anticipatory anxiety during the delivery of the first inhalation that is attenuated or absent during the delivery of the second inhalation. In other words, the first inhalation will induce an anxiogenic response, regardless of gas content (this overall would be stronger in the gas condition due to the added effect of 7.5% CO_2_). When CO_2_ is delivered first, this would exacerbate the anxiogenic effect of the gas thereby increasing the difference between conditions. In contrast, when air is delivered first, this will produce an anxiogenic response in the control condition that reduces the difference between the conditions. For walking speed, the slowing effect of CO_2_ was only evident in the CO_2_/air group. This explanation can be further supported if similar patterns are observed in the subjective and physiological data. However, we only observed a gas-by-order interaction for DBP, indicating that some other factors may be contributing the effects of order on gait variables.

More broadly, this study supports the claim that gait is altered by psychological states. It replicates previous work showing changes in gait as a function of high and low mood states (Sloman et al., 1987), although the qualitative nature of these changes differed in our study. There is a paucity of literature in this area, and inconsistency in methods and measures makes it difficult to draw conclusions regarding the qualitative differences (or similarities) across states. Our data identify changes in gait when people are in heightened anxious states that may translate to anxiety disorders as they are characterised by higher frequency, duration and/or severity of anxiety response, and this should be investigated in future research. The mechanisms underlying gait changes in other psychological disorders such as depression are unclear and are likely not mediated through anxiety. The importance and timeliness of exploring the relationship between gait and psychological states is exemplified by the technological advances in remote sensing. This passive monitoring of motor behaviour could act as a marker for the onset or maintenance of psychiatric episodes in real world environments, which could aid diagnosis and treatment. Further work is required to identify reliable motoric signatures for different psychological disorders and states, but our work supports the previous findings that suggest these signatures exist and are measurable.

One limitation that should be acknowledged is that we are unable to differentiate the effects of CO_2_-induced anxiety from other effects of hypercapnia. For example, it is possible that the changes to movement which we observed were not mediated by anxiety, but may be due to changes caused by increased inhalation of CO_2_ that are independent of the anxiogenic effects. It is noteworthy that we did not observe general reductions in movement during CO_2_ inhalation (turning behaviour increased in this condition), suggesting that there was not a general dampening of motor activity due to lower oxygen levels for example. There are limited studies investigating the effects of hypercapnia on movement. Animal studies have reported mixed findings with evidence of both altered sensory–motor activity (JavadiEsfahani & Kwong, 2019) and no movement effect (Branigan, Elkhalifa, & Pamenter, 2018) to increased environmental carbon dioxide. One human study explored the effect of experimentally manipulating inspired gas concentrations of oxygen (O_2_) and CO_2_ on walking behaviour (Kinkead, Bach, Johnson, Hodgeman, & Mitchell, 2001). Unlike our sustained inhalation model, this was done on a step-by-step basis with the aim to explore whether blood gas receptors (sensitive to O_2_ and CO_2_-related change) were sensitive to energy costs and altered movement accordingly. While the procedure was deemed effective, gait seemed to be unaffected by the manipulation with individuals showing perseverance of normal walking patterns. These results arguably suggest limited gait impact of low-level hypercapnia, supporting that effects may be more reliable at higher doses that are associated with anxiety. However, this is speculative and requires more investigation. In future studies, it may be possible to statistically explore whether motoric effects are mediated by changes in subjective or physiological anxiety, but we were not sufficiently powered to explore this in the current study.

Taken together, these findings suggest that during a 7.5% anxiogenic challenge, individuals make adjustments to their locomotive behaviour. Our data suggest that individuals walk more slowly and rotate their body more when navigating through an aperture. This may be due to greater caution when faced with obstacles in an anxious state. These observable differences in locomotor behaviour as a function of emotional state offer support to the idea that advancing sensing technology may beused to infer emotion states from observation of motor behaviour in naturalistic settings.

## Electronic supplementary material

Below is the link to the electronic supplementary material.

Supplementary material 1 (DOCX 217 kb)
