# Supplementary material for: Effects of state anxiety on gait: a 7.5% carbon dioxide challenge study
Source: Psychol Res. 2020 Jul 31;85(6):2444–52. doi: 10.1007/s00426-020-01393-2 (PMC8357656; doi:10.1007/s00426-020-01393-2)
Supplement: Supplementary file 1 — Supplementary material 1 (DOCX 217 kb) [file 426_2020_1393_MOESM1_ESM.docx]

**Supplementary Information: Effects of state anxiety on gait: a 7.5% carbon dioxide challenge study**

**S1. Methods**

S1.1 Participant exclusion criteria

Exclusion criteria included pregnancy or breastfeeding, recent use of illicit drugs, daily smoking, high caffeine (≥8 drinks per day) or alcohol (greater than 35 alcoholic units/week if female or 50 alcoholic units/week if male) consumption, uncorrected visual or auditory impairment, balance problems, recent use of prescribed medication (past 8 weeks), asthma, history of migraine, respiratory illness, cardiovascular disease and history of drug/alcohol dependence. Pregnancy and recent drug use were verified by urine screen while all other criteria were assessed via self-report. Participants were also required to be in good psychiatric health, which was assessed using an adapted version of the MINI-International Neuropsychiatric Interview (Sheehan et al., 1998). Participants were also excluded if they had high blood pressure (>140/90 mmHg), bradycardia (<50 beats per minute), tachycardia (>90 beats per minute), or body mass index (BMI) outside of a healthy range (<17 or >30 kg/m^2^), all of which were physically assessed. Participants were asked to refrain from consuming alcohol for 36 hours before the session. Expired breath alcohol was measured upon arrival and participants were excluded if their breath alcohol reading was higher than zero.

S1.2 Setting apertures

Apertures were created by the researcher placing two poles at various points along the laboratory on each trial. The poles had a flat, round base of ~1 cm. thick, a diameter of ~15 cm, and a central pole that was 2.2 m. high. The poles were placed at either near (5.5 m) or far (7.5 m) from start of walkway. These apertures were either located towards the left or right side of the laboratory, with the left apertures having their outer (left pole) 1.15 m from the left wall and the right apertures having their outer (right) pole 1.10 m from the right wall. The wall-to-wall width of the laboratory is 4.6 m, so the effective width of the “walkable” area in the experiment was approximately 2.4 m. The position of these apertures varied across trials using a pre-determined pseudo-random sequence of the four positions (left near, right near, left far, left near), with each position repeated 5 times for each inhalation. This sequence was generated independently for each participant. The width of the aperture was tailored to each participant, using a appeture to shoulder ratio of 1.2.

S1.3 Defining and measuring gait variables

Markers and the sternum rigid body were labelled using the ‘Automated Identification of Markers’ model in Qualisys Track Manager (QTM, Qualisys, Göteborg, Sweden). The local coordinate system of the rigid body on the chest was aligned with the global coordinate system of the room (y facing forward, x facing right and z is up). The primary axis of rotation was defined to be the z-axis, as we are primarily interested in the turning angle (i.e. yaw). Gaps of up to 292 ms were filled using spline interpolation in QTM. Using custom-written software in Matlab, we selected the 6DOF (degrees-of-freedom) data from the rigid body in a spatial window of -4 to +4 m. relative to the y-position of the aperture (defined as the average y-coordinate of the two poles). The position of the poles themselves were defined as the median x and y coordinates recorded between approximately 1-4 s into the trial. As the poles do not change

position, the recorded values fluctuate minimally and the precise epoch over which their position is extracted, is not critical.

Speed and angular rotation of the body were computed from the same set of markers fixed to a plate worn on the sternum. Figure 2B and C shows that there is some noise in these data, but because both measures were averaged in 2 m sections (effectively a form of low-pass filtering), we did not filter the marker position and orientation data. We first tested whether the very first position sample of the sternum plate (in y) was within 0.25 m of the start of our Region of Interest (ROI = 8 m. centered on the aperture). If the gap between the first y-sample and the start of the ROI was larger than this value, we rejected the trial. We adopted a similar criterion on the gap between the last sample and the end of the ROI. The criterion of 0.25 was based on previous measurements that indicated that a gap of this size could be interpolated with sufficient accuracy to keep the data. If the data were kept (i.e. the recording covered the full ROI or only minimally fell short), we tested the remaining samples for any gaps that had been missed by the QTM software. Our criterion for filling a gap was that the size of the gap in space was less than 0.25 m and the duration should be less than 300 ms. Again, these thresholds were adopted from previous measurements that indicated we could accurately interpolate such gaps (Ludwig et al., 2018). We used spline interpolation with data from the entire available trajectory (within the ROI). Interpolation was performed separately for x and y coordinates, as a function of time. Whenever a gap was found that met our criteria for filling, the trajectory with the gap and its filled section was shown to the experimenter, who then had the opportunity to reject or keep the trial based on a visual check.

**S2. Results**

S2.1 Order effects

*Entry point:* There was a weak gas by order interaction for entry point (*F*(1,20)=3.36, *p*=0.08, η_g_=.01) (see Figure S1). This interaction is shown by the cross-over in the dashed lines of Figure S1: the within-subject effect of CO_2_ was larger for the group that received this treatment first (magenta line).


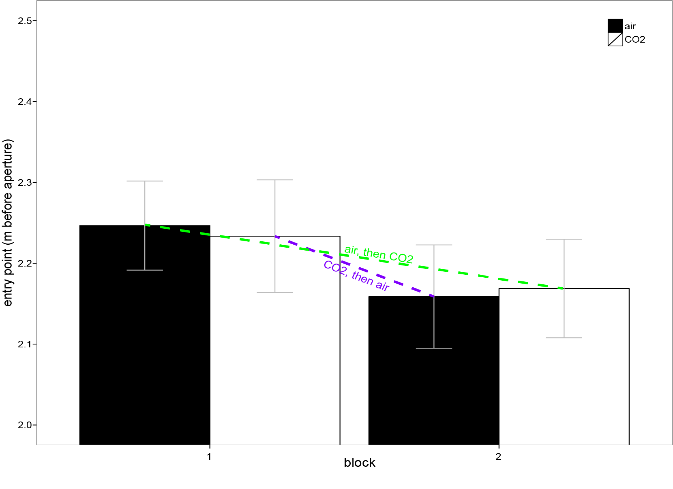


*Figure S1.* Entry point as a function of block (i.e., inhalation one versus inhalation two) and condition. Error bars are within-subject standard errors of the mean (Morey, 2008). Note: for any one block, the comparison between air and CO_2_ is a between-subjects comparison. Across blocks, bars with different colours correspond to the same subjects (i.e. the within-subject comparison, shown by dashed lines and labels).

*Walking speed:* There was a main effect of order (*F*(1,20)=4.14, *p*=0.06, η_g_=.15) and an order by condition interaction (*F*(1,20)=5.81, *p*=0.03, η_g_=.02). In Figure S2, the main effect of order is illustrated by the dashed lines (air-CO_2_ group) lying above the solid lines. The magnitude of the interaction was small but is shown by the larger effect of CO_2_ on velocity for the group that received the treatment first (in Figure S2, the difference between the solid lines is larger than the difference between the dashed lines). However, note that the *within-subject* comparisons (compare lines of the same type across the two panels) are consistent with a slower walking speed in the CO_2_ condition for both gas orders.


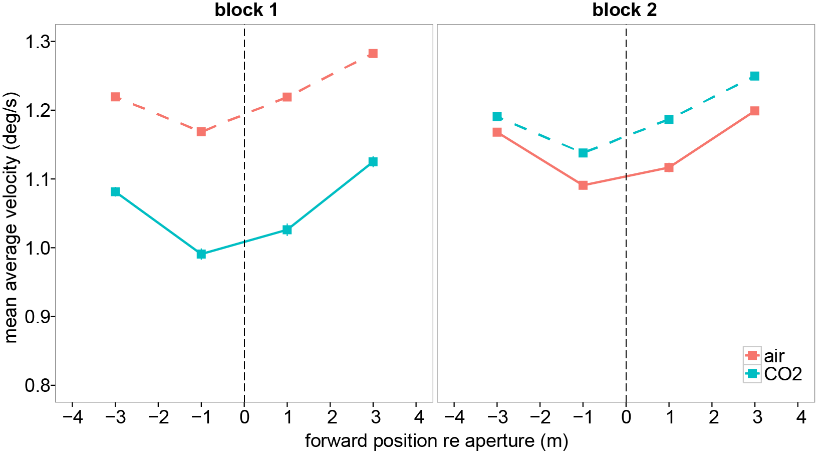


*Figure S2.* Walking speed, separately for the two blocks. The aperture position is marked with the vertical dashed line. The line type (solid v dashed) indicates the two groups of participants. Error bars represent within-subject standard errors of the mean (but, in many cases are smaller than the plotting symbol).

*Body orientation (through an aperture):* There was a main effect of order (*F*(1,20)=11.03, *p*=0.003, η_g_=.11), with greater body rotation in the CO_2_/air group compared to air/CO_2_ group.

*Subjective and cardiovascular outcomes:* There was a gas by order interaction for DBP (*F*(1,20)=3.74, *p*=0.068, ηp^2^=.16), with greater difference between gas conditions in the CO_2_/air order (Air: *M =* 72.6, *SE* = 2.0; CO_2_: *M* = 77.7, *SE* = 1.9) compared to the air/CO_2_ order (Air: *M =* 73.1, *SE* = 1.8; CO_2_: *M* = 73.7, *SE* = 1.8). There were no clear main effects of order (*ps* > .27) or order by gas interactions (*ps* > .15).

S2.2 Bayes Factors

*Entry point:* A Bayes Factor ANOVA resulted in four models, beyond the basic, null effect model. The Bayes Factors for these four models relative to the null, were all below 1, ranging from 0.004 (full model that contains condition, order and their interaction) to 0.21 (model with only order as a predictor). Taken together, there is no evidence for the role of any of these variables in modulating the entry point.

*Walking speed:* With three factors (condition, bin, order), the number of possible linear models is large (i.e., 18), and therefore we restricted analysis to models that were of *a priori* interest (see Table 1). All models have a “subject” random factor (i.e. a subject-specific mean across all conditions) and the null model only contains the subject as random effect. We test to what extent more complex models that include predictor variables other than subject, increase the marginal likelihood of the data.

In the case of velocity, all models included in Table S1 are much more “likely” than the null model. The full model that includes all three predictors and their two and three-way interactions shows the highest Bayes Factor. A direct comparison between the full model and the next best model (Condition + Bin), resulted in a Bayes Factor of 6.3e+23 in favour of the full model.

Table S1. Bayes Factor comparisons with a null model that contains only a subject-specific mean as a random factor. Values greater than 1 indicate support for the more complex model listed here.

| **Model** | **BF_10_ (velocity)** | **BF_10_ (turning behaviour)** |
| --- | --- | --- |
| Condition | 1.66e+67 | 1.39 |
| Bin | 5.63e+127 | 4.83e+12 |
| Condition + Bin (main effects only) | 1.29e+211 | 7.28e+12 |
| Condition * Bin (main effects and interaction) | 3.40e+208 | 8.43e+10 |
| Full (all main effects and all two and three-way interactions) | 6.41e+234 | 1.02e+6 |

*Body rotation (through an aperture):* The same Bayes Factor analysis was applied as for walking speed and are included in Table S1. For this variable, the model with condition and bin as predictor variables (but not their interaction) was the best model overall. However, the Bayes Factor, relative to the next best model (bin only as predictor) was only 1.61, indicating that in this analysis there is little evidence in favour of the main effect of gas condition across the entire Region of Interest.

**References**

Ludwig, C. J. H., Alexander, N., Howard, K. L., Jedrzejewska, A. A., Mundkur, I., & Redmill, D. (2018). The influence of visual flow and perceptual load on locomotion speed. *Atten Percept Psychophys, 80*(1), 69-81. doi: 10.3758/s13414-017-1417-3

Sheehan, D. V., Lecrubier, Y., Sheehan, K. H., Amorim, P., Janavs, J., Weiller, E., . . . Dunbar, G. C. (1998). The Mini-International Neuropsychiatric Interview (M.I.N.I.): the development and validation of a structured diagnostic psychiatric interview for DSM-IV and ICD-10. *J Clin Psychiatry, 59 Suppl 20*, 22-33;quiz 34-57.
